# Impact of vaccination on measles, mumps, and rubella antibody titers in Japanese healthcare workers: An observational study

**DOI:** 10.1371/journal.pone.0230329

**Published:** 2020-03-24

**Authors:** Taku Ogawa, Takashi Inoue, Kei Kasahara, Mitsuru Konishi, Keiichi Mikasa

**Affiliations:** 1 Center for Infectious Diseases, Nara Medical University, Nara, Japan; 2 Institute for Clinical and Translational Science, Nara Medical University, Nara, Japan; 3 Center for Health Control, Nara Medical University, Nara, Japan; Public Health England, UNITED KINGDOM

## Abstract

Given the complicated history of Japan’s National Immunization Program, a significant proportion of Japanese people including healthcare workers (HCWs) still lack adequate immunity against measles, mumps, and rubella (MMR), resulting in occasional outbreaks. In 2014, the Japanese Society of Infection Prevention and Control (JSIPC) published vaccination guidelines for HCWs. We evaluated antibody titers before and after MMR vaccination in HCWs at the Nara Medical University Hospital, the attainment rate of the target antibody titers defined by the JSIPC guidelines, and the safety of vaccines. We measured MMR antibody titers in HCWs, followed by inoculation with the respective monovalent vaccines and/or trivalent MMR (tMMR) vaccine according to the JSIPC guidelines. Among 467 HCWs evaluated, antibody titers against measles and mumps measured using the IgG-enzyme immunoassay increased from 11.0 [interquartile range (IQR): 8.0–13.6] to 13.7 (IQR: 11.3–16.9; *P* < 0.001) and from 2.8 (IQR: 2.1–3.5) to 4.8 (IQR: 3.7–5.7; *P* < 0.001), respectively. By evaluating a logarithmic value of log_2_(X + 1) converted from an antibody titer X, antibody titers against rubella measured using the hemagglutination assay increased from 3.2 (IQR: 0–4.1) to 6.0 (IQR: 4.6–8.0; *P* < 0.001). Antibody titer elevated following tMMR vaccination was lower than that following monovalent vaccination in a single dose of the measles-containing, a single dose of the mumps-containing, and two doses of rubella-containing vaccine groups (*P* = 0.01, 0.01, and <0.001, respectively). After vaccination, 20.0%, 61.5%, and 46.2% of HCWs attained target antibody titers specified by the JSIPC guidelines for measles, rubella, and mumps, respectively. The systemic response in female HCWs who underwent monovalent mumps vaccination was statistically higher than that in others. Although the vaccination program for HCWs according to the JSIPC guidelines caused increased MMR antibody titers, the rates of attaining the target criteria were low.

## Introduction

The measles–mumps–rubella combined vaccine (tMMR) was introduced in Japan in 1989. However, the side effect of aseptic meningitis due to the mumps component became a problem, and in 1993, this vaccine was practically discontinued. Since then the Japanese government has become reluctant toward routine vaccination. The measles–rubella combined vaccine (bMR) revived a routine vaccination in 2006. Since 2008, the government has adopted a policy of providing catch-up immunization opportunities to those who have lost the chance of vaccination but has not yet achieved sufficient herd immunity. As a result, local outbreaks of measles, rubella, and mumps as well as the occurrence of congenital rubella syndrome have been recently reported in Japan. [1–3].

Healthcare workers (HCWs) need to respond to such outbreaks and are thus expected to be more likely to contract these infections than the general population. Therefore, it is necessary for HCWs to ensure that they are immune against measles, mumps, and rubella. Under such circumstances, guidelines for HCW vaccination were required. Thus, the Japanese Society of Infection Prevention and Control (JSIPC) prepared vaccine guidelines for HCWs in 2009 and revised them in 2014 [4].

The JSIPC guidelines basically recommend confirming two written vaccination histories of each HCW for measles, rubella, and mumps. When written vaccination histories are not available, JSIPC guideline recommends evaluating the antibody titer of HCWs to determine how to perform additional vaccination. Indeed, performing two doses of additional vaccination without any antibody titer examination are acceptable. Several antibody titer measuring methods are mentioned in the JSIPC guidelines. IgG-enzyme immunoassay (EIA), particle agglutination (PA), and neutralization test are recommended for measles; hemagglutination inhibition (HI) and IgG-EIA for rubella; and IgG-EIA for mumps. When measles IgG-EIA value is <2.0, rubella HI value is <1:8, and mumps IgG-EIA value is <2.0, the antibody titer is thought to be negative. On the other hand, when measles IgG-EIA value is ≥16.0, rubella HI value is ≥1:32, and mumps IgG-EIA value is ≥4.0, the antibody titer is thought to be positive. When antibody titers of HCWs are not in any of the categories, they are defined as intermediate. When the antibody titer is in intermediate category, HCWs are recommended to receive one more dose of vaccination with written record. When antibody titer is negative, they are advised to be inoculated two more doses of vaccine. The aim of this research was to assess affection on antibody titer, how much HCWs can achieve the criteria for sufficient immunity, and how many side reactions will occur if vaccination is performed according to the JSIPC guidelines. Furthermore, we aimed to evaluate the seropositivity of each disease before vaccine intervention.

### Study design and vaccination implementation program

**Fig 1:**
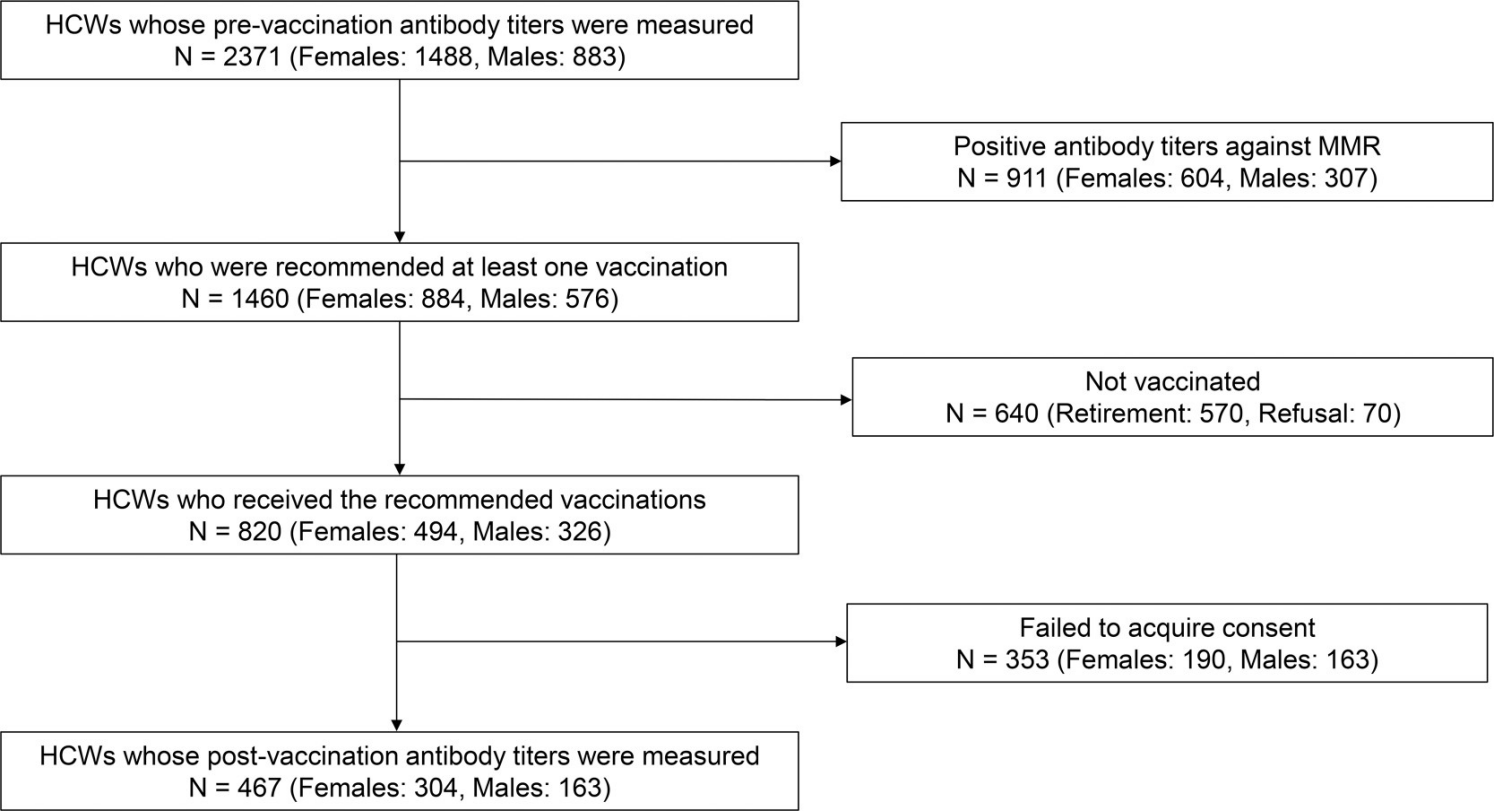
Flow chart detailing subject selection in the vaccination implementation program and this study.

This study was performed as part of the vaccination implementation program conducted in Nara Medical University Hospital (NMUH) that aimed to administer necessary vaccinations to HCWs. NMUH vaccine implementation program was compliant with JSIPC vaccine guidelines for HCWs issued on 2014. Fig 1 summarizes the subject selection process in the JSIPC guidelines and the study. First, antibody titers against MMR were measured as part of the routine medical check-up program for all HCWs (n = 2,371) during October 2014–December 2014. Antibody titers were measured using the IgG-enzyme immunoassay (IgG-EIA) kit for measles and mumps and the hemagglutination assay (HI) kit for rubella (Denka Seiken Co., Ltd., Japan). The results were then classified into three categories (negative, intermediate, and positive), as indicated in Table 1. At NMUH, the IgG-EIA method is used to evaluate antibody titers against measles and mumps, whereas the HI method is used for rubella. Both methods are recommended in the JSIPC guidelines. HCWs who submitted a history of two documented vaccinations were not required to receive vaccinations regardless of their antibody titer. Even if the antibody titer is “intermediate” or “negative,” HCWs with two written records of vaccination history were not required to be vaccinated. If HCWs did not submit a history of two documented vaccinations, they were required to receive one vaccination dose if their antibody titer was intermediate and two doses if their antibody titer was negative. Vaccination was not mandatory, and HCWs were allowed to refuse vaccination for any reason. However, HCWs received vaccination if they were unsure of having received two doses earlier. Both trivalent MMR (tMMR) vaccine and the respective monovalent vaccines were used; tMMR vaccine was preferred over multiple monovalent vaccines for those who required more than two different vaccinations, although HCWs could choose which vaccines to receive. When the study was conducted, simultaneous administration of multiple vaccines was not a common practice in Japan [5], and we thought there would be an increasing risk of mistaking correct vaccine for others; therefore, they were separately administered at least 27 days apart. Vaccinations were administered between April 2015 and February 2016. Follow-up antibody titer tests were performed between October 2016 and December 2016.

**Table 1:** Criteria for antibody titers against measles, mumps, and rubella in the 2014 JSIPC guidelines [4].

	Measurement method	Negative	Intermediate	Positive
**Measles**	IgG-EIA	<2.0	≥2.0, <16.0	≥16.0
**Rubella**	HI	<1:8	≥1:8, <1:32	≥1:32
**Mumps**	IgG-EIA	<2.0	≥2.0, <4.0	≥4.0

EIA, enzyme immunoassay; HI, hemagglutination assay

In March 2015, a letter documenting necessary vaccination and timing was sent to 1,460 HCWs who required vaccinations. Simultaneously, a letter asking cooperation with this study was also included. This study required participants (1) to have their antibody titers measured using a blood test at the following year’s medical check-up (between October 2016 and December 2016, no additional blood drawing was necessary for this study) to determine “post-vaccination” titers and (2) to submit information regarding the side effects of the received vaccination. Finally, of 820 HCWs who received the recommended vaccinations, 467 participated in this study. This study was approved by the Ethics Committee of NMUH (authorization number: 1400) and was in compliance with the Japanese domestic law and the Declaration of Helsinki.

### Vaccines used in this study

The following vaccines were used in this study: Priorix® (GlaxoSmithKline) as the tMMR vaccine, Dried Live Attenuated Measles Vaccine (Takeda Pharmaceutical Co., Ltd.) as the monovalent measles vaccine (mMeV), Dried Live Attenuated Rubella Vaccine (Kitasato Daiichi Sankyo Co., Ltd.) as the monovalent rubella vaccine (mRuV), and Dried Live Attenuated Mumps Vaccine (Takeda Pharmaceutical Co., Ltd.) as the monovalent mumps vaccine (mMuV). The description of each vaccine is as follows: tMMR: Schwartz strain (≥10^3^ CCDI50) as measles, Wistar RA 27/3 strain (≥10^3.7^ CCDI50) as rubella, and RW 4385 strain (≥10^3^ CCDI50) as mumps derived from the Jeryl Lynn strain; mMeV: Schwarz FF-8 strain of ≥5000 CCDI50; mRuV: Takahashi strain ≥1000 PFU; and mMuV: Torii strain ≥5000 CCDI50. All the details of vaccines were obtained from the package insert. As tMMR vaccine is unapproved in Japan, we used the tMMR vaccine manufactured in Belgium, which was affirmatively transported in an appropriate temperature-controlled supply chain. The use of tMMR vaccine was approved by the Ethics Committee of NMUH (authorization number 660).

### Safety of the inoculated vaccines

We collected data on adverse events (AEs) that occurred after vaccination. Using questionnaires, the participants responded regarding whether there were adverse reactions after vaccinations. We investigated AEs in terms of local reactions (e.g., pain, swelling, and redness of the inoculation site lasting ≥2 days) and systemic reactions (e.g., fever, headache, nausea and vomiting, and skin symptoms other than inoculation sites). All participants were instructed to freely fill details if there were symptoms other than those mentioned above. Then nature of AEs listed in the free entry column (as local or systemic reactions) was determined by two or more physicians at our center after consultation. Furthermore, participants were asked if there was a need to abstain from work because of AEs.

### Statistical analysis

The population analyzed in this study for the efficacy verification and safety assessment of the vaccines included 467 HCWs who had their antibody titers measured after providing written consent among 820 HCWs who had been recommended to receive vaccination. The target number of HCWs was considered to be adequate because antibody titer elevation could be statistically verified in at least 44 HCWs, assuming that the effect size of titer elevation (negative to intermediate or intermediate to positive) was 2.0 and the standard deviation of the titers was a maximum of 2.0 with a two-sided significance level of 0.05 and a power of 0.90. All statistical analyses in this study were performed using EZR Ver.1.36 (Saitama Medical Center, Jichi Medical University, Japan; http://www.jichi.ac.jp/saitama-sct/SaitamaHP.files/statmedEN.html; Kanda, 2012), which is a graphical user interface for R (The R Foundation for Statistical Computing, Vienna, Austria, Version 2.13.0). More precisely, EZR is a modified version of the R commander (Version 1.6–3) that was designed to add statistical functions frequently used in biostatistics [6]. All P-values were two-sided, and P-values of ≤0.05 were considered statistically significant.

For the statistical analysis on the rubella antibody titer X determined using HI, a logarithmic value of log_2_(X + 1) was used. We used the Wilcoxon signed rank-sum test to determine statistically significant differences between antibody titers before and after vaccinations. The resulting significance was confirmed by a multiple linear regression analysis with the antibody titer as an objective variable and the pre-inoculated antibody titers, age, and sex as explanatory variables. Among HCWs who received one vaccination, the elevation in antibody titers was compared between the monovalent vaccine (mV)-inoculated group and the tMMR-inoculated group using the Mann–Whitney U test. The resulting significance was confirmed by a multiple linear regression analysis with post-vaccinated antibody titers as the objective variable and with the objective groups for comparison as a dichotomous explanatory variable while adjusting for pre-vaccinated antibody titer, age, and sex. Among HCWs who were vaccinated twice, we compared the increase in antibody titers between the mV + mV group and the one tMMR + one mV group using the Mann–Whitney U test. The resulting significance was confirmed by multiple linear regression analysis with post-vaccination antibody titer as the objective variable and with the objective groups for comparison as a dichotomous explanatory variable while adjusting for age and sex.

The antibody titer target achievement level was evaluated as the percentage of HCWs who achieved the criteria set in this study. For vaccine safety assessment, we statistically determined the difference in AE prevalence in each vaccine group. Using the MMR-vaccinated group as a reference, we examined AE incidence in the other vaccine groups using a multivariate logistic regression analysis, in which the objective dichotomous variable was the presence or absence of AEs and the explanatory variable was the type of vaccine (four vaccine groups), while adjusting for age and sex. Moreover, we evaluated AE frequency for each vaccine using the Fisher’s exact test stratified by sex. AEs that required HCWs to be hospitalized or to be absent from their work were individually explained because the number of such AEs was small.

## Results

### Participants’ demographic characteristics

Table 2 summarizes the characteristics of HCWs included in the vaccination implementation program (left column, HCWs who had their antibody titers measured before vaccination, n = 2371) and the participants of this study (right column, HCWs who received vaccination and whose antibody titers were measured before and after vaccination, n = 467). The age and female to male ratios were comparable between the two groups. As the participants comprised HCWs with at least one negative or intermediate MMR antibody titer, the proportion of HCWs with negative or intermediate MMR antibody titers was higher in the study participants. Table 3 displays details on the types of negative or intermediate antibody titers (left column), necessary doses of vaccines (middle column), and the number of HCWs who were inoculated (right column). Vaccines were selected and administered based on this table.

**Table 2:** Demographic characteristics and disposition of participants.

Variables	Participants of the vaccination implementation program (n = 2371)				Participants whose antibody titers were evaluated (n = 467)			
**Age (years)**								
** Median**	38				38			
** IQR**	30–48				31–47			
**Sex (n%)**								
** Female**	1488		62.8%		303		64.9%	
** Male**	883		37.2%		164		35.1%	
**Negative or intermediate antibody titer (n%)**								
** Measles**	896		37.8%		289		60.1%	
** Rubella**	466		19.7%		143		30.6%	
** Mumps**	755		31.8%		225		48.1%	
**Age/sex distribution (n%)**								
**Age**	Female		Male		Female		Male	
**≤24**	43	2.9%	10	1.1%	4	1.3%	2	0.7%
**25–29**	336	22.6%	128	14.5%	62	20.5%	20	6.6%
**30–34**	238	16.0%	156	17.7%	56	18.5%	35	11.6%
**35–39**	203	13.6%	144	16.3%	39	12.9%	33	10.9%
**40–44**	192	12.9%	135	15.3%	51	16.8%	29	9.6%
**45–49**	183	12.3%	84	9.5%	37	12.2%	15	5.0%
**50–54**	143	9.6%	69	7.8%	27	8.9%	11	3.6%
**55–59**	87	5.8%	75	8.5%	18	5.9%	8	2.6%
**60–64**	41	2.8%	55	6.2%	7	2.3%	9	3.0%
**≥65**	22	1.5%	27	3.1%	2	0.7%	2	0.7%

IQR: inter quartile range

**Table 3:** Combinations of negative or intermediate antibody titers in 467 HCWs and inoculated vaccines.

Types of negative or intermediate antibody titers			Inoculated vaccines and dose				Inoculated number of HCWs		
Measles	Rubella	Mumps	tMMR	mMeV	mRuV	mMuV	Total	Female	Male
△				1			141	92	49
	△				1		30	25	5
	×				2		23	11	12
		△				1	76	51	25
		×				2	20	13	7
△	×		1		1		7	5	2
×	△		1	1			1	1	0
△	△		1				26	22	4
△		×	1			1	16	10	6
△		△	1				68	32	36
	×	×	2				4	1	3
	△	×	1			1	3	3	0
	△	△	1				13	13	0
△	×	×	2				1	1	0
△	△	×	1			1	7	4	3
△	×	△	1		1		5	4	1
△	△	△	1				23	16	7
							467	304	163

Positive: (blank); Intermediate: △; Negative: ×; HCWs, healthcare workers; tMMR: trivalent measles–mumps–rubella; mMeV: monovalent measles vaccine; mRuV: monovalent rubella vaccine; mMuV: monovalent mumps vaccine; HCWs: Healthcare workers.

### Seroprevalence

**Fig 2:**
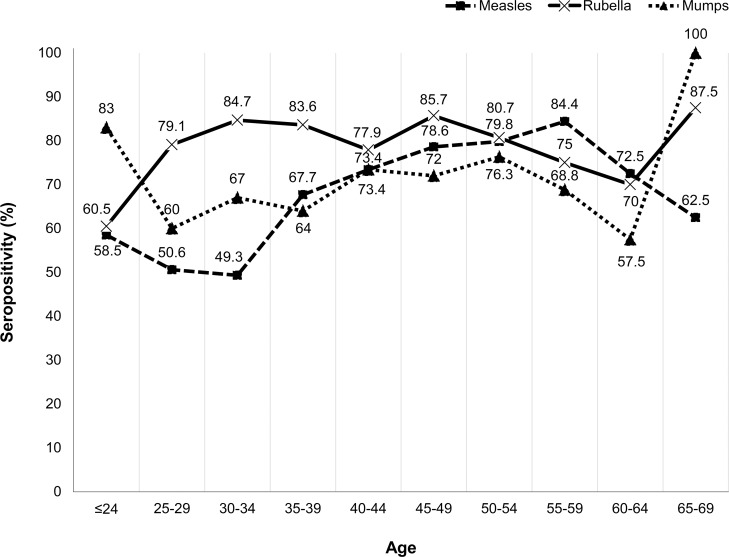
Seropositivity ratio by age groups (females).

**Fig 3:**
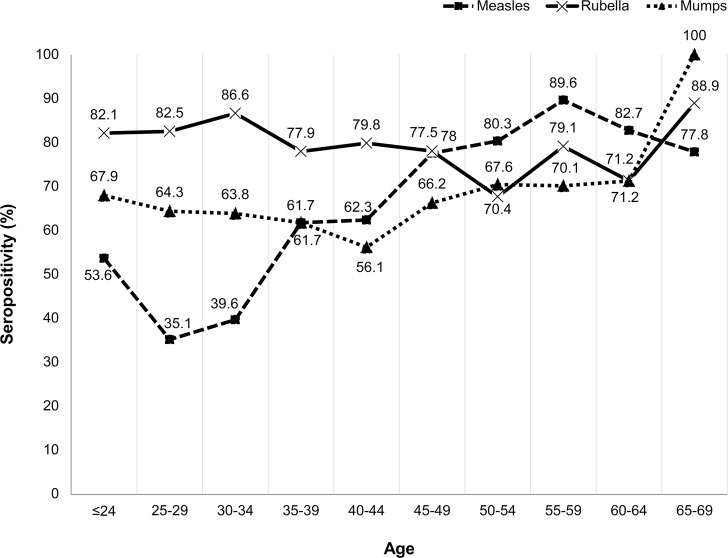
Seropositivity ratio by age groups (males).

Figs 2 and 3 describe the seroprevalence of MMR based on the JSIPC guidelines by classification according to sex and age (five-year increments). Overall, the seropositivity of measles was the lowest and that of rubella was the highest in both males and females. The seropositivity of measles increased with age until 50–59 of age; however, it decreased in participants aged 60–64 and 65–69 years.

### Antibody titer elevation and seropositivity after vaccination

The average antibody titer of HCWs with negative or intermediate measles antibody titers was 11.0 [interquartile range (IQR): 8.0–13.6], which increased to 13.7 (IQR: 11.3–16.9; *P* < 0.001) after vaccination. Regarding rubella, the antibody titer increased from 3.2 (IQR: 0–4.1) pre-vaccination to 6.0 (IQR: 4.6–8.0; *P* <0.001) after vaccination. Regarding mumps, the antibody titer increased from 2.8 (IQR: 2.1–5.5) to 4.8 (IQR: 3.7–5.7; *P* < 0.001) (Table 4).

**Table 4:** Antibody titer response to the entire vaccination protocol.

	Measles	Rubella	Mumps
**HCWs with insufficient titer (n)**	281	143	225
**Pre-vaccination titer [titer (IQR)]**	11.0 (8.0–13.6)*	3.2 (0–4.1)*	2.8 (2.1–5.5)*
**Post-vaccination titer (titer [IQR])**	13.7 (11.3–16.9)*	6.0 (4.6–8.0)	4.8 (3.7–5.0)

HCWs, healthcare workers, IQR, interquartile range

*P < 0.001

Among 281 HCWs who received measles-containing vaccines, only one had negative antibody titer and 280 had intermediate antibody titers; however, only 56 (19.9%) achieved a positive antibody titer after vaccination (Table 5), and the remaining HCWs had intermediate antibody titers despite the net increase in antibody titers, as described in Table 4. Among 143 HCWs who received rubella-containing vaccines, 88 (61.6%) achieved positive antibody titers after vaccination. Interestingly, although 95.3% (41/43) of HCWs with negative antibody titers attained positive antibody titers after vaccination, only 47% (47/100) of HCWs with intermediate antibody titers achieved positive antibody titers after vaccination. Among 225 HCWs who received mumps-containing vaccines, 43.5% attained positive titers after vaccination. Additionally, 31.1% and 46.9% of HCWs with negative and intermediate antibody titers, respectively, achieved positive titers after vaccination.

**Table 5:** Effect of vaccines on achieving cut-off values in HCWs with negative or intermediate antibody titers.

Measles			Post-vaccination		Total
		Positive	Intermediate	Negative	
	Intermediate	52	228	0	280
**Pre-vaccination**	Negative	0	1	0	1
	Total	52	229	0	281
**Rubella**			Post-vaccination		Total
		Positive	Intermediate	Negative	
	Intermediate	38	62	0	100
**Pre-vaccination**	Negative	41	2	0	43
	Total	79	64	0	143
**Mumps**			Post-vaccination		Total
		Positive	Intermediate	Negative	
	Intermediate	90	87	2	179
**Pre-vaccination**	Negative	14	31	1	46
	Total	104	118	3	225

### Comparison of antibody titer elevation among vaccine types

Among HCWs inoculated with one vaccination dose, antibody titer evaluation was compared between the mV-inoculated group and the tMMR-inoculated group after adjustment for age and sex (Table 6). Measles and mumps had significantly lower antibody titers in the tMMR group (*P* < 0.001 for both). We compared the mV + mV group and the one tMMR + one mV group among HCWs who were vaccinated twice; the elevation in antibody titers was low for rubella antibody titers in the latter group (*P* < 0.001). Considering these results, the antibody titer elevation by tMMR vaccine was lower than that by the corresponding monovalent vaccine.

**Table 6:** The relevance of the antibody titer increment effect and vaccine type.

	Measles	Rubella	Mumps
**HCWs whose titer measured**	N = 281	N = 143	N = 225
**One vaccination dose inoculated group**	N = 280	N = 100	N = 180
**mV group (antibody titer elevation, IQR)**	N = 134	N = 30	N = 73
	2.5 (1.2–5.2)*	1.0 (0–1.0)#	1.8 (1.3–2.7)*
**tMMR group (antibody titer elevation, IQR)**	N = 146	N = 70	N = 107
	0.9 (−0.3–2.8)*	0.9 (0–1.0)#	0.3 (−0.2–0.9)*
**Two vaccination doses inoculated group**	N = 1	N = 43	N = 45
**mV + mV group (antibody titer elevation, IQR)**	N = 0	N = 23	N = 19
	−	8.0 (7.0–8.5)*	3.3 (3.2–4.1)##
**One tMMR + one mV group (antibody titer elevation, IQR)**	N = 1	N = 20	N = 26
	11.7 (11.7)	6.0 (6.0–7.0)*	3.1 (2.6–5.3)##

HCWs, healthcare workers; IQR: interquartile range; mV: monovalent vaccine; tMMR: trivalent measles–mumps–rubella

*P < 0.001

# P = 0.038

## P = 0.32

### Incidence of adverse effects

In the analysis of systemic and local reactions by vaccines, age was not a confounding factor for either systemic or local reactions whereas sex was a confounding factor only for local reactions (*P* = 0.050). Thus, AE incidence in each vaccine was determined by stratifying with regard to sex (Table 7). In the Fisher’s exact test, the omnibus test revealed a significant difference among females for systemic reactions (*P* = 0.043) whereas the post-hoc pairwise test found significantly higher systemic reactions with mMuV than with tMMR vaccine (P = 0.030). Because no male HCWs experienced a systemic response, we could not compare the incidence of systemic reactions between each vaccine group among males. Based on the Fisher’s exact test, no significant difference was noted in the incidence of local reactions to vaccines in both males and females (*P* = 0.72 for both, Table 7).

**Table 7:** Correlation between vaccine type and adverse event frequency.

	Male			Female		
	Vaccine type	Incidence (%) (n)	Omnibus comparison	Incidence (%) (n)	Omnibus comparison	Pairwise comparison
**Systemic reaction**	tMMR	0% (0)	NA	1% (1)	P = 0.043	P = 0.03
	mMeV	0% (0)		2.8% (1)		
	mRuV	0% (0)		1% (1)		
	mMuV	0% (0)		7.8% (5)		
**Local reaction**	tMMR	9.4% (6)	P = 0.72	19.1% (20)	P = 0.72	
	mMeV	11.8% (2)		16.7% (6)		
	mRuV	14.3% (7)		13.3% (12)		
	mMuV	6.5% (2)		14.1% (9)		

tMMR: trivalent measles–rubella–mumps; mMeV: monovalent measles vaccine; mRuV: monovalent rubella vaccine; mMuV: monovalent mumps vaccine

Only two HCWs reported taking few days off from work or attending a hospital because of AEs. One of these was a 42-year-old female who received single-dose tMMR vaccination because of intermediate antibody titer against rubella and measles. She developed submandibular gland swelling 1 day after vaccination. Consequently, she had to visit a nearby general practitioner and took a day off from her work. The other HCW was a 31-year-old female who received single-dose mMeV vaccination because of the absence of measles antibody titer; she developed a fever of >38°C and had to take 3 days off from work. Fever developed within 14 days after vaccination; however, it was not known exactly when the fever developed.

## Discussion

Although several national surveillance analyses on seropositivity have been conducted, it is often difficult to directly compare seropositivity owing to differences in the assays and cut-off values employed in the surveillance. For example, the Japanese national surveillance on measles antibody seropositivity conducted using the PA method with a cut-off of 1:16 titers showed a seropositivity rate of approximately 95% in all age groups >2 years [7]. Kanamori et al. reported a seropositivity rate of 95.5% among HCWs in their hospital using the measles ELISA kit used in the present study; however, their cut-off value (positive, ≥4.0) was lower than our cut-off value (positive, ≥16.0) [8]. Our seropositivity results were lower than these results, particularly in HCWs aged <35 years; however, our results were interpreted based on the higher cut-off proposed by the 2014 JSIPC guidelines. Regarding the seropositivity rate of rubella, a national surveillance conducted in 2017 using HI with a cut-off of 1:32 revealed a seropositivity rate of approximately 70%–80%, which is similar to our results [9]. Males born between 1962 and 1979 (age 36–53 in this study) had few chances of receiving rubella vaccines in Japan [10]; thus, the lower rubella antibody titers observed in males aged 35–54 years in this study may reflect this situation. Regarding mumps, we found no particular difference between the result of research conducted between 2012 and 2013 using the same method and the same cut-off value (EIA, ≥4.0) [11]. To the best of our knowledge, our study is the first to describe the seropositivity of MMR among HCWs as per the 2014 JSIPC vaccination guidelines. The results form the basis of future recommendations regarding who should receive vaccination, particularly among HCWs.

A strength of our study is that we evaluated the impact of each vaccine on the increase in antibody titer after vaccination and the achievement of positive antibody titers. Although a statistically significant increase was observed for MMR, the mean post-vaccination titer did not reach the positive range for measles or rubella, which resulted in a low rate of attaining the positive criteria defined by the 2014 JSIPC guidelines. Targeted HCWs may have included individuals who may had innately low antibody responses to vaccination; nevertheless, our result is important because it reflects the real situation in which the target population includes adult HCWs who are required to have higher immunity against MMR. Yoshioka et al. reported the significance of herd immunity gained by a vaccination program for HCWs [12]. It is unclear whether the statistically significant increase in antibody titers with a low target attainment rate is clinically significant; however, we should bear in mind that setting a goal of reaching the positive range may require some HCWs to receive more than two vaccination doses. It is also unclear whether attainment rates increase after receiving more than two vaccination doses. Lee et al. reported that the minimal measles-specific IgG concentration necessary to prevent symptomatic measles was approximately 500 mIU/mL, which is equivalent to 10.9 according to our measuring method [13]. Among HCWs whose antibody titers were measured post-vaccination, 298 (63.8%) had negative or intermediate measles antibodies. If the cut-off value was lowered to 10.9, the number of HCWs with negative or intermediate measles antibody titer would have decreased to 167 (35.8%).

Among those who received rubella vaccination, 41 of 43 HCWs with negative titers attained positive titers after vaccination; however, only 47 of 100 HCWs with intermediate titers attained positive titers after vaccination. This means that HCWs with lower rubella titers were more likely to achieve positive titers; it is difficult to find a reasonable explanation for this result. One possibility is that two rubella vaccinations in HCWs with negative titers may be far more efficacious than one rubella vaccination in HCWs with intermediate titers. The antibody titers of two HCWs changed from intermediate to negative despite receiving mumps-containing vaccine. Both had antibody titer of 2.2 by IgG (EIA) before vaccination and less than 2.0 after vaccination. This change in titer may have been due to measurement errors or poor response to the vaccine. Among HCWs whose antibody titers are intermediate before and after inoculation, there are some people who show no changes in antibody titers. These events are in principle considered to be of the same phenomena.

Interestingly, there was a statistical difference in the increase in the antibody titers between the mV- and tMMR vaccine-inoculated groups in the single-dose inoculated group for measles and mumps and two-dose inoculated groups for rubella. Again, it is unclear whether this statistical difference is clinically significant; however, it must be considered that the effect may differ according to the vaccination administered. Although the difference may be attributed to vaccines, it can also be attributed to the difference between the groups vaccinated. These differences may include the following: (1) HCWs missed multiple vaccinations during their childhood, (2) they were less exposed to these infections, and (3) they were inherently less responsive to vaccines. Conversely, HCWs who received monovalent vaccines had at least one of the other infections, indicating that HCWs had received vaccinations or were exposed to the disease.

This study suggests that systemic reactions are markedly higher in the female mMuV group compared with the other vaccine groups; this observation contradicts previous Japanese studies. However, in each case, AE severity was not high; moreover, each case reportedly recovered from symptoms in a few days. We adjudicated that these AEs are endurable. In addition, tMMR vaccine (Priorix^®^) can be safely used in Japanese adults. Incidentally, in this study, AE incidence of the vaccines was lower than that in previous studies [14,15]. We think that this is due to HCWs taking low AEs after vaccination for granted and not reporting them.

This study has some limitations. First, vaccination history documentation is often lost or not even recorded in Japan. Reportedly, people tend to have an inaccurate memory of their vaccination and morbidity history [16]. Hence, it was challenging to precisely collect vaccination and morbidity histories. Second, the background of 860 HCWs who were vaccinated and agreed or disagreed to participate in the study may have differences. People who agreed to participate in this study may have been concerned about not acquiring adequate antibody titers because they did not have a vaccination history; this may lead to low and poor elevation of antibody titers. Finally, although we noted the vaccination of 1460 people, only 820 people could be vaccinated. This fact has to be considered as a problem for the feasibility of our vaccine program. We anticipate that the biggest reason for this difference is that NMHU is a public hospital and has undergone many personnel changes. Furthermore, it remains unclear why 70 people refused vaccination on their own initiative.

Although our vaccination program was planned according to the JSIPC guidelines, the proportion of HCWs whose antibody titer after vaccination were positive were not so high. This was particularly apparent in measles. As pointed out in another study [17], we think that the cut-off value of the antibody titer in JSIPC might lead to unnecessary vaccine administration to HCWs and should thus be reviewed in the future. Immunization against MMR is affected not only by the antibody titer but also by the avidity of the antibody [18,19]. Reportedly, determining the avidity of antibodies is useful as a method to differentiate between nonimmune individuals and individuals with failure of secondary vaccine [20,21]. There has been a report of HCW infection with measles despite two vaccination records [22]. Based on these facts, we believe that there are limitations to the JSIPC guidelines that recommend estimating true immune status using only conventional methods, such as antibody titer and the number of vaccinations. The plaque reduction neutralization test (PRNT) has been reported to be a method that can more accurately evaluate immunity to infectious diseases than the IgG (EIA) method [23]. Because PRNT is expensive and is conducted in limited facilities, it is not practical to be adapted to daily practice of infection control at this moment. However, on a long-term basis, we think that a novel evaluation method such as PRNT might be considered in the future guideline development.

## Conclusions

To the best of our knowledge, this is the first study to evaluate a vaccination program based on the 2014 JSIPC guidelines by evaluating the elevation and rate of achieving target antibody titers and the safety of vaccination. Although substantial increases in antibody titers were safely attained, the rates of achieving the target criteria specified by the JSIPC guidelines were low, particularly in measles. The efficacy of antibody titer evaluation may differ according to the vaccines used. Further studies are necessary to establish more proper indicators of immunity and protocol for vaccinating HCWs.
